# Predicting Severity and Intrahospital Mortality in COVID-19: The Place and Role of Oxidative Stress

**DOI:** 10.1155/2021/6615787

**Published:** 2021-03-26

**Authors:** Ivan Cekerevac, Tamara Nikolic Turnic, Nevena Draginic, Marijana Andjic, Vladimir Zivkovic, Stefan Simovic, Romana Susa, Ljiljana Novkovic, Zeljko Mijailovic, Marija Andjelkovic, Vladimir Vukicevic, Tatjana Vulovic, Vladimir Jakovljevic

**Affiliations:** ^1^Department of Internal Medicine, Faculty of Medical Sciences, University of Kragujevac, Serbia; ^2^Clinic for Pulmonology, Clinical Center Kragujevac, Kragujevac, Serbia; ^3^Department of Pharmacy, Faculty of Medical Sciences, University of Kragujevac, Serbia; ^4^Department of Physiology, Faculty of Medical Sciences, University of Kragujevac, Serbia; ^5^Clinic for Cardiology, Clinical Center Kragujevac, Serbia; ^6^Department of Infectious Diseases, Faculty of Medical Sciences, University of Kragujevac, Serbia; ^7^Clinic for Infectious Diseases, Clinical Center Kragujevac, Serbia; ^8^Department of Biochemistry, Faculty of Medical Sciences, University of Kragujevac, Serbia; ^9^Center for Laboratory Diagnostics, Clinical Center Kragujevac, Serbia; ^10^Center for Anesthesiology and Resuscitation, Clinical Center Kragujevac, Serbia; ^11^Department of Surgery, Faculty of Medical Sciences, University of Kragujevac, Serbia; ^12^Department of Hyman Pathology, IM Sechenov First Moscow State Medical University, Moscow, Russia

## Abstract

SARS-CoV-2 virus causes infection which led to a global pandemic in 2020 with the development of severe acute respiratory syndrome. Therefore, this study was aimed at examining its possible role in predicting severity and intrahospital mortality of COVID-19, alongside with other laboratory and biochemical procedures, clinical signs, symptoms, and comorbidity. This study, approved by the Ethical Committee of Clinical Center Kragujevac, was designed as an observational prospective cross-sectional clinical study which was conducted on 127 patients with diagnosed respiratory COVID-19 viral infection from April to August 2020. The primary goals were to determine the predictors of COVID-19 severity and to determine the predictors of the negative outcome of COVID-19 infection. All patients were divided into three categories: patients with a mild form, moderate form, and severe form of COVID-19 infection. All biochemical and laboratory procedures were done on the first day of the hospital admission. Respiratory (*p* < 0.001) and heart (*p* = 0.002) rates at admission were significantly higher in patients with a severe form of COVID-19. From all observed hematological and inflammatory markers, only white blood cell count (9.43 ± 4.62, *p* = 0.001) and LDH (643.13 ± 313.3, *p* = 0.002) were significantly higher in the severe COVID-19 group. We have observed that in the severe form of SARS-CoV-2, the levels of superoxide anion radicals were substantially higher than those in two other groups (11.3 ± 5.66, *p* < 0.001) and the nitric oxide level was significantly lower in patients with the severe disease (2.66 ± 0.45, *p* < 0.001). Using a linear regression model, TA, anosmia, ageusia, O_2_^−^, and the duration at the ICU are estimated as predictors of severity of SARS-CoV-2 disease. The presence of dyspnea and a higher heart rate were confirmed as predictors of a negative, fatal outcome. Results from our study show that presence of hypertension, anosmia, and ageusia, as well as the duration of ICU stay, and serum levels of O_2_^−^ are predictors of COVID-19 severity, while the presence of dyspnea and an increased heart rate on admission were predictors of COVID-19 mortality.

## 1. Introduction

SARS-CoV-2, a new RNA virus, caused the worldwide pandemic in 2020 by developing severe acute respiratory syndrome [1]. Coronavirus disease 2019 (COVID-19) is induced by SARS-CoV-2 with varying symptomatology, from being asymptomatic to having pneumonia of different degrees of severity of acute respiratory distress syndrome (ARDS) or death [2]. Some reports from China estimated that the majority of cases were limited to a mild and moderate symptomatology, noticed in 81% of the infected population, 14% patients with progressive, severe pneumonia and 5% of the infected population developing ARDS [3]. And while the mortality rate still cannot be estimated, in most cases, it is related to multiple organ failure and ARDS. Direct or indirect lung injury results in the acute systemic inflammatory response and leads to ARDS, the same as acute myocardial injury and renal injury (7–17% and 3–15%, respectively) [2, 4–7]. However, the most important pathophysiological processes that lead to severe forms of the disease are not yet precisely known.

It has been postulated that marked elevation of proinflammatory cytokines and cytokine storm are significant contributors to the disease progress, as they significantly correlate to the severity and COVID-19 mortality [8]. Besides cytokine release and elevations in classic markers of acute inflammation, infiltration of immune cells, and progressive lymphopenia, the particularly ratio of neutrophil-to-lymphocyte is recognized as a prognostic marker [9]. It is hypothesized that in COVID-19, this infiltration of neutrophils leads to reactive oxygen species (ROS) secretion that boosts both hyperinflammation and further damage [10]. Alongside, the disturbed antioxidant-prooxidant balance that leads to oxidative stress (OS) is also contributed by the decreased antioxidant defense in viral infections, leading to lipid peroxidation and DNA oxidation [11].

However, besides several reviews hypothesizing the potential role of oxidative stress in COVID-19 and its possible implication in the disease severity, there is no data available on prooxidative and antioxidative parameters and their possible effects on prognosis in COVID-19 patients. Therefore, we aimed to explore their possible role in predicting severity and intrahospital mortality of COVID-19, alongside with other laboratory and biochemical procedures, symptoms, and admission arterial gas analysis parameters.

## 2. Patients and Methods

### 2.1. Ethical Concerns

The study was approved by the Ethical Committee of Clinical Center Kragujevac, number 01/20/485 from 24/04/2020. All researched procedures were done in Clinical Center Kragujevac, Serbia, and in the Laboratory for Cardiovascular Research, Faculty of Medical Science. In the study, all procedures were done according to the Declaration of Helsinki (Revision 2013) and Good Clinical Practice. Written informed consent for participation was obtained from all patients.

### 2.2. Protocol of the Study

This study was designed as an observational prospective cross-sectional clinical study which was conducted on 127 patients with diagnosed respiratory COVID-19 viral infection. All participants were included in the study during the second peak of the pandemic period from April to the end of August 2020. Inclusion criteria were written informed consent, older than 18 years, and PCR-confirmed (polymerase chain reaction test) SARS-CoV-2-etiology of disease. All patients were, after admission, followed-up for different periods, according to the course of the disease. From all patients, we collected anamnestic data, clinical symptom data, and biochemical data and oxidative stress parameters on the first day of hospitalization. We set two primary goals in our study:

1. To determine the predictors of severity of COVID-19 and disease progression
2. To determine the predictors of negative outcome (fatal) or positive outcome (complete or incomplete regression on chest roentgenogram at hospital discharge)

### 2.3. Clinical Management

All confirmed COVID-19 patients were hospitalized with precautions for airborne transmission. Patients were followed prospectively during hospital treatment. All predictors were determined on the first day of hospitalization: clinical symptoms and signs, comorbidities, and biochemical and oxidative stress parameters.

Patients with moderate to severe hypoxia (requiring of inspired oxygen ≥ 40%) were transferred during hospitalization to the intensive care unit (ICU) for high-flow oxygen via nasal cannula, noninvasive ventilation, or invasive mechanical ventilation. According to the World Health Organization (WHO), patients were assigned to one of the three categories/groups [12]:

1. Patients with a mild form of COVID-19 (mild symptoms up to mild pneumonia) (*n* = 17)
2. Patients with a moderate COVID-19 (dyspnea, hypoxia, or less than 50% lung involvement on imaging) (*n* = 40)
3. Patients with a severe COVID-19 (patients with severe respiratory failure, need for high flow oxygen therapy, mechanical ventilation, sepsis, or multiorgan system dysfunction) (*n* = 70)

### 2.4. Biochemical Analysis

All biochemical procedures were done on the first day of hospital admission in a specialized biochemical laboratory of the Clinical Center Kragujevac, Serbia. Complete blood cell count (CBC) was measured using a hematology analyzer (DxH 800 Hematology Analyzer by Beckman Coulter). The biochemical parameters such as glucose, creatinine, urea, cholesterol, triglyceride (TG), aspartate aminotransferase (AST), alanine aminotransferase (ALT) gamma-glutamyl transferase (GGT), lactate dehydrogenase (LDH), total and direct bilirubin, C-reactive protein (CRP), sodium, and potassium were estimated from the serum samples by using standard kits in an automatic clinical chemistry analyzer (AU680 Clinical Chemistry Analyzer by Beckman Coulter). Measurement of the vitamin D level was performed using an automated immunoassay analyzer—the Alinity i system (Abbott Laboratories, IL, USA) that utilizes the chemiluminescent microparticle immunoassay (CMIA) principle. The level of procalcitonin in the serum was determined by the method of electrochemiluminescence, on the immunochemistry analyzer (Cobas e 411 by Roche). D dimer concentration measurement was performed on coagulation analyzer ACL-TOP 300 (Instrumentation Laboratory, Bedford, USA) employing the automated latex-enhanced particle immunoturbidimetric method.

### 2.5. Determination of Markers of Oxidative Stress in Plasma and Lysate Samples

In plasma samples, on the first day of hospital treatment, we measured the concentration of prooxidative markers such as superoxide anion radical (O_2_^−^), hydrogen peroxide (H_2_O_2_), nitric oxide (NO^−^), and the index of lipid peroxidation measured as TBARS (TBARS). The determination of the nonenzymatic antioxidant activity, such as the activity of the enzymatic defense system, by evaluating the catalase (CAT) and concentrations of superoxide dismutase (SOD) and reduced glutathione (GSH) was determined in the lysate.

Determination of the superoxide anion radical (O_2_^−^) was performed by measuring the concentration of the superoxide anion radical (O_2_^−^) after the reaction of nitro blue tetrazolium in Tris buffer with the plasma at 530 nm. Distilled water solution served as a blank probe [13]. An indirect method for monitoring nitric oxide (NO) by determining nitrate (NO_3_^−^) and nitrite (NO_2_^−^) was performed as previously described by Pick and Keisari [14]. The plasma volume of 0.5 ml was precipitated with 200 *μ*l of 30% sulfosalicylic acid, then vortexed for 30 min, and centrifuged at 3000 × *g*. Supernatant and Griess reagent in the equal volumes containing 0.1% naphthalene ethylenediamine dihydrochloride/1% sulphanilamide in 5% phosphoric acid were added and then incubated for 10 min in the dark and measured at 543 nm [15]. The degree of lipid peroxidation in the plasma (TBARS) was estimated by measuring TBARS using 1% thiobarbituric acid in 0.05 NaOH, incubated with plasma at 100°C for 15 min, and measured at 530 nm. Distilled water served as a blank probe [16].

The level of reduced glutathione (GSH) was determined based on GSH oxidation with 5.5-dithio-bis-6.2-nitrobenzoic acid using the method of Beutler [17]. CAT activity was determined according to Aebi. Lysates were diluted with H_2_O (1 : 7 *v*/*v*) and treated with chloroform-ethanol (0.6 : 1 *v*/*v*) in order to remove the hemoglobin. After that, 1 ml of 10 mM H_2_O_2_, 100 *μ*l of the sample, and 50 *μ*l of CAT buffer were added to the samples. Detection was performed at 360 nm [18]. In order to determine the SOD activity, the epinephrine method of Beutler was used. A total of 1 ml of carbonate buffer and 100 *μ*l of lysate were mixed, after which100 *μ*l of epinephrine was added. Detection was performed at 470 nm [19].

### 2.6. Statistical Analysis

Statistical analysis was conducted with the SPSS for Macintosh version 26.0 software. Data are presented as the mean values ± standard errors of the mean/standard deviations with statistical significance. For the categorical variable, results are presented as the frequency from the total sample (in percent). The normality of the distribution of the parameters being analyzed was determined using the Shapiro-Wilk test. We used a parametric Friedman's ANOVA test or a nonparametric Kruskal-Wallis test or chi-squared test according to the data characteristics and distribution. Also, Pearson's correlation analysis and the linear regression model were used to test the association between variables and to find the significant predictors for general outcome and severity of SARS-CoV-2 infection. The accepted level of significance was defined as *p* < 0.05 for confidence interval of 95%.

## 3. Results

### 3.1. Demographic and Clinical Characteristics of Patients Infected with SARS-CoV-2

In Table 1, the basic demographic characteristics and presence of comorbidity in the study population according to the subgroups of patients are shown. Distribution of comorbidities and hypertension was significantly different in three groups of COVID-19 patients. Comorbidities were present in 66.7% of the patients with severe COVID-19 infection and in 20.5% of the patients with a moderate form of the disease. One of them, hypertension, was present in 70.1% of the patients with a severe form and in 16.4% of the patients with a moderate form of COVID-19 infection (Table 1). Other comorbidities were not significantly different in our three groups.

In Table 2, distribution of specific symptoms of COVID-19 infection in the study group is shown. It is observed that elevated body temperature, cough, and diarrhea were significantly different, distributed in these three groups: the elevated temperature was significantly present in the group with a severe form of the disease (58.6%) and in the group with a moderate form of the disease (28.8%); cough was present in 61.5% of the patients with a severe form and 23.1% of the patients with a moderate form of the disease; diarrhea was not present in 58.4% of the patients with a severe form and 31.9% in the group of patients with a moderate form of the disease (Table 2). Other specific symptoms such as anosmia, ageusia, and dyspnea were not significantly different, present in mild, moderate, and severe groups.

### 3.2. Respiratory and Cardiovascular Symptoms of COVID-19 in the Study Population

In our study, we evaluated the means of respiratory and cardiovascular symptoms in three groups of patients (Table 3). Patients with severe forms of infection were significantly older than other patients in groups. Also, respiratory and heart rates at admission were significantly higher in patients with severe and mild forms compared with respiratory and heart rates in patients with a moderate SARS-CoV-2 form of the disease. Also, SBP (systolic blood pressure) and DBP (diastolic blood pressure) were significantly higher in patients with the severe form of the disease. Other respiratory signs such as SatO_2_ (oxygen saturation) did not significantly differ between these groups (Table 3).

Furthermore, from all observed hematological and inflammatory markers, only WBC (white blood cell) count and LDH activity were significantly different in patients with mild, moderate, and severe forms of the disease (Table 4).

### 3.3. Prooxidative and Antioxidative Parameters in Blood in SARS-CoA-2 Infection

In Table 5 are presented values of the main prooxidants and antioxidative enzymes measured in our study at hospital admission. We have observed that in the patients with a severe form of SARS-CoV-2, levels of superoxide anion radicals were significantly higher than those in the two other groups. On the other hand, the nitric oxide level was significantly lower in patients with severe COVID-19. Catalase activity was significantly lower in the patients with a moderate form of the disease compared with the group of patients with a severe form of the disease (Table 5).

### 3.4. Correlation and Linear Regression Analysis of Data

**Figure 1:**
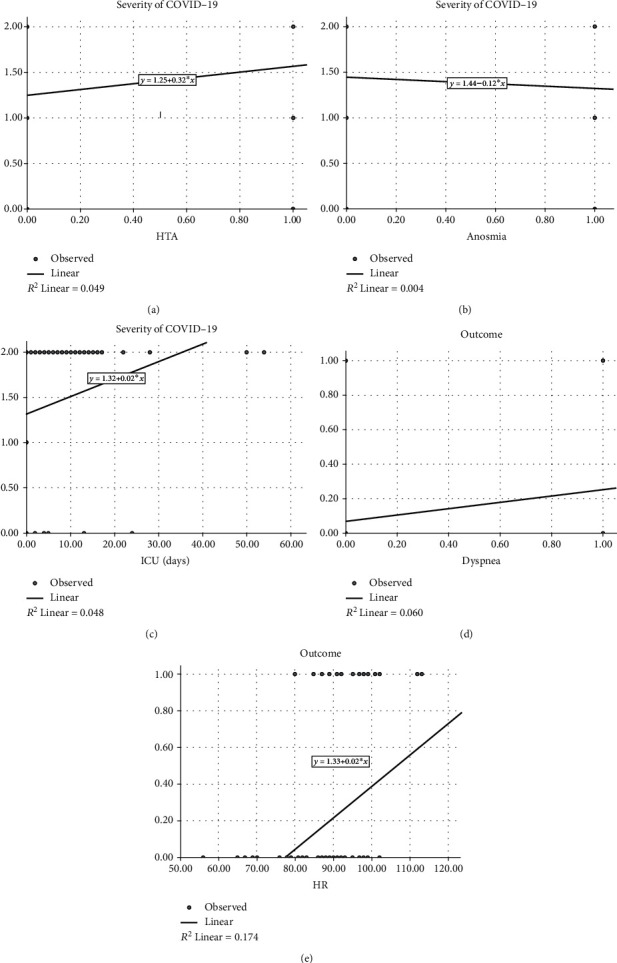
(a-e) Regression variable plots.

In correlation analysis (Table 6), we included all statistically significant variables from previous statistical research. We observed the positive correlation between age and most of the tested variables (Table 6). Also, HTA as a categorical variable was significantly associated with RR (respiration rate), SBP, DBP, WBC, and negative outcome (fatal outcome). In our study from total number of patients, fatal outcome was observed in 14.4% and positive outcome was in 85.6%. Furthermore, anosmia showed a strong positive correlation only with ageusia. Dyspnea was in positive moderate correlation with RR, negative outcome (fatal), and RTG outcome. On the other hand, RR was in inverse correlation with NO^−^ levels and in positive correlation with HR, SBP, DBP, negative outcome, and duration at the ICU. Also, the mean duration at the ICU was in correlation with the severity of the disease, so, the duration in the group with a severe form of the disease was 5.96 ± 4.2 days and that in the group of with mild COVID-19 was 3.06 ± 2.2 days. In general, the duration of hospital treatment was 14.4 ± 5.2 days in the mild group, 10.45 ± 5.6 in moderate, and 16.62 ± 9.2 in patients with a severe form. Interestingly, concentrations of NO^−^ were in moderate negative correlation with CAT activity, RTG outcome, duration at the ICU, and in-hospital death (Table 6).

the linear regression model for two separated dependent variables, such as the general outcome (positive or negative) and severity of COVID-19 infection, provided significant results (Tables 7 and 8 Figures 1(a)–1(e)). HTA, anosmia, ageusia, O_2_^−^, and duration at ICU are estimated as predictors of severity of COVID-19 (Table 7). Regression variable plots present the nature of association of two different variables (Figures 1(a)–1(e)). As it shows, HTA was in positive linear association with severity of COVID-19 infection, as well as the duration of stay in the ICU. Also, dyspnea was in positive linear association with positive outcome, while the anosmia was in negative linear association with severity of SARS-CoV-2 infection in patients. Very interestingly, HR was in positive linear association with outcome in patients and the higher heart rate was a good predictor of negative outcome in patients with confirmed COVID-19 disease (Figures 1(a)–1(e)). Definitely, the presence of dyspnea and the higher heart rate were confirmed as predictors of a negative outcome (fatal) (Table 8).

## 4. Discussion

The main purpose of this clinical prospective cross-sectional study was to provide novel information about potential molecular mechanisms during the different degree of COVID-19 in adult patients and in consequence to provide potential new preventive and therapeutical strategies.

Our population of 127 patients consisted predominantly of middle-aged, nonsmoker males, with hypertension as the most common comorbidity. Distribution of comorbidity and hypertension was significantly different in the mild, moderate, and severe groups. Also, hypertension was present in 70.1% of the patients with a severe form and in 16.4% of the patients with a moderate form of COVID-19 infection.

First reports from COVID-19 showed a higher incidence of hypertension in patients hospitalized with severe COVID-19 [7, 12, 20].

The link between COVID-19 and hypertension remains ambiguous, despite these observations. The severity of COVID-19 disease is amplified in aging members of the population with a higher prevalence of hypertension; on the other hand, it corresponds to a percentage in the general population. Still is unknown, whether this relationship is age-associated or causally linked to obesity and diabetes mellitus. We also found a higher incidence of diabetic and obese patients in the group of severe COVID-19 patients, but without statistical significance.

Despite the fact that it has been reported that current smokers show the expression of the ACE-2 gene which is higher comparing to nonsmokers and the possibility of a higher risk for COVID, all epidemiological data that have been published so far show low prevalence of smokers, as well as the lack of a link between current smoking status and severity of COVID-19 [13, 14]. Furthermore, there was no link between patients who never smoked and ex-smokers and severity of COVID-19. Also, the frequency of current smokers in our study did not differ considerably between groups with different severity of COVID-19. The number of active smokers in the study group was low 11/127 (8.6%), given that the prevalence of smoking in Serbia stands around 35%. Literature data suggests that smoking status, however, appears to correlate with ACE2 gene expression thus implicating differences in gender-specific behaviors. A previous study compared current smokers with never smokers and concluded that smokers have significantly upregulated ACE2 expression in the lung and oral epithelium [21].

We found that cough as a symptom of respiratory infection, was a significantly more common symptom in patients with severe COVID-19. In a study by Leung et al., fatigue, expectoration, and stuffed nose were prognostic symptoms of severe COVID-19. Fever existed in 92.1% of the COVID-19 patients but was not predictor of disease severity [22]. On other hand, in a study by Guan et al., fever was significantly correlated with the trend of intensification of COVID-19. In this study, despite that 43.8% of the patients have fever upon admission, 88.7% of the patients had a fever in the course of hospitalization [6]. Fever that developed as a result of critical pulmonary infection was regularly observed in patients with severe COVID-19; therefore, temperature monitoring should not be the only screening method for COVID-19.

Relevant studies have shown that elderly patients have an increasing risk of serious diseases, with 80% of deaths occurring in individuals over 60 years old [2]. This association is believed to be related to the weakened immune function in the elderly population [23]. However, the rate of the disease severity increased with age, suggesting variable susceptibility to the virus in different age groups, not only related to the weakened immune function of the elderly population. Our results also show that the mean age was highest in the severe COVID-19 group (61.39 ± 12.87). Despite this, age-related associations need further investigation. Also, several social, behavioral, and comorbid factors are implicated in the generally worse outcomes in men compared with women. Underlying biological sex differences and their effects on COVID-19 outcomes still are not clear [21].

Analyzing vital signs, we found in the group with severe COVID-19 a significantly higher respiratory rate, heart rate, and values of systolic and diastolic pressure at admission, while SatO_2_ values did not significantly differ at admission. It has been shown that the increased respiratory rate and decreased oxygen saturation were associated with higher odds of mortality [24]. Also, the available data shows that COVID-19 patients who experienced the start of CPAP, NIV, admission to ICU, or death in the hospital at admission were increasingly tachypneic and required growing amounts of supplemental oxygen. On average, these patients suffered a minor increase in heart rate [25]. These results correspond to the results of Zhou et al., where tachycardia was a rare feature [7].

When lymphocyte and leukocyte counts were compared between the patients with severe versus mild cases of COVID-19, we found a significantly higher number of WBC in the group with severe COVID-19, while the number of lymphocytes did not vary significantly between groups. Overall, patients with severe COVID-19 tend to have lower lymphocyte counts and higher leukocyte counts. It is not yet known why lymphopenia is associated with a severe form of COVID-19. The possible association is explained by the consequences of direct infections with lymphocytes, apoptosis caused by inflammation, lactic acidosis that inhibits lymphocytes, and destruction of lymph tissue [26]. In our study baseline, LDH values were highest in the group with severe COVID 19. Li et al. analyzed the relationship between LDH and disease progression and in-hospital death. They showed that LDH levels in the group with fatal outcome were substantially higher. A cutoff LDH value of 353.5 U/l predicted the in-hospital mortality (sensitivity of 94.4% and a specificity of 89.2%) [27]. The high LDH level was a risk factor for the progression of diseases in mild COVID-19 patients [28]. It has been reported that LDH may be a predictor of respiratory failure in patients with COVID [29], as well as a predictive factor for early recognition of severe COVID-19 cases [30].

The onset of inflammation was considered to have a primordial role in the evolution of the disease. The group of severe COVID-19, in our study, had the highest mean value of CRP at admission although there was no statistical significance between the groups. During inflammatory disease phases of infection, CRP can activate the immune system classical complement cascade and modulate phagocytic cell activity [31]. In COVID-19, it has been reported that CRP levels can be used for early identification of pneumonia [27] and the assessment of severe pulmonary infectious diseases [32], even though the exact effect remains unclear. In addition, our findings show that CRP levels on admission were an early indicator for COVID-19 severity, which is consistent with recent publications [33]. Previous authors suggested that an early elevation in C-reactive protein (CRP) greater than 15 mg/l provides a marker of disease severity and levels greater than 200 mg/l on admission are independently associated with five times the odds of death [21].

In our study, anosmia was singled out as a predictor of a milder form of the disease by linear regression analysis (Table 6, Figure 1(e)). Patients with anosmia have been shown to have lower mortality (OR: 0.438) and less severe disease, with lower ICU admission as well. [34]. A potential explanation is a different inflammatory response in these patients and distinctive clinical presentation. Moreover, some of the studies even point that the persistence of severe chemosensitive dysfunction could be related to the need for hospitalization after 20 days. Up to this day, there are no studies which analyze other contributing risk factors for the outcomes [35].

We have a few concerns about our results, particularly those linked to arterial hypertension. In our study, these comorbidities significantly affected the severity of the disease during hospital treatment (Figure 1(a)). Guan et al. found that in the critical ill group (admission to ICU, the use of mechanical ventilation, or death) (35.8% versus 13.7%) with severe forms of the COVID-19 disease (23.7% versus 13.4%), hypertension was a more prevailing condition [6]. It has also been described that cardiovascular diseases and hypertension were more frequent in patients with fatal outcome than those who were discharged (43% versus 28%, *p* = 0.07) [36, 37]. All the evidence seem to be consistent. Contributing factors such as older age as well as associated cardiovascular disease, primarily coronary heart disease, can significantly affect the association between arterial hypertension and severity of COVID-19 and mortality rate.

The length of stay in the ICU significantly affected the severity of the disease in our study (Figure 1(c)). The mean value of days spent in the ICU in the group with severe COVID-19 was 5.96 ± 4.2 days, while the duration of hospital treatment was 14.4 ± 5.2 days in the mild group, 10.45 ± 5.6 in the moderate group, and 16.62 ± 9.2 in patients with a severe form. It has been reported that the majority of the patients (58%) were admitted to the ICU on the first day of hospital admission and in the following two days for most cases [38]. In severe pneumonia, late admission to ICU was associated with poor outcome [33]. The high extent of ICU stays and the duration of stay make challenges for hospital management. The main challenge is adequate initial assessment and treatment of patients to shorten their stay in the ICU and duration of hospitalization. On the other hand, it has been shown that a longer stay in the ICU leads to a better outcome. Reasons include slower patients weaning from the ventilator, longer follow-up of patients, prevention of relapse, and readmission in the ICU [39].

Although symptoms like dyspnea are subjective and tend to increase with reduced lung function or age, it often signifies serious underlying disease. We have shown that dyspnea on hospital admission represents an independent predictor of negative outcome (fatal). In the study of Xie et al., dyspnea was independently related with fatal outcome in COVID-19 (hazard ratio, 2.60, *p* = 0.01) [35]. Results of meta-analysis, based on 11 studies with 2091 COVID-19 patients, showed that dyspnea was frequently correlated with higher mortality (OR = 4.34, *p* < 0.001) [40].

Regarding the redox status, we evaluated the concentrations of reactive oxygen and nitrogen species, as well as the activity of enzymes of the antioxidative defense system in COVID-19 patients. As we know, the pathophysiology of pneumonia (bacterial or viral) includes activation of defense mechanisms, characterized by an immense influx of activated phagocytes [41]. Also, we know that the polymorphonuclear neutrophils and macrophages kill microorganisms by using ROS, increasing oxidative stress in the lung, which may cause a direct injury in the tissue and activate transcription factors. This leads to a local inflammatory response, which could progress to a systemic inflammatory response [42]. Definitely, oxidative stress could be an important factor in pneumonia and the crucial pathogenic mechanism underlying the development of this respiratory disease. In our study, we confirmed that in the severe form of SARS-CoA-2, levels of superoxide anion radicals were considerably higher than those in the two other groups. On the other hand, levels of nitric oxide were significantly lower in patients with the severe COVID-19. Catalase activity was significantly lower in the moderate form of the disease compared with the severe form (Table 5). A very interesting result was that the O_2_^−^ as of the measured markers could be a sign of the severity of SARS-CoV-2 disease (Table 7). Also, the bioavailability of NO^−^ was in negative correlation with CAT activity, RTG outcome, duration at ICU, and in-hospital stay in general.

Today, there are very limited data about the role of oxidative stress in different degrees of viral pneumonia caused by SARS-CoV-2. Up to date, there are just a few studies which examined the redox status in COVID-19 patients. Laforge et al. examined the association of reactive oxygen species (ROS) with COVID-19 disease severity and concluded that ROS induce tissue damage, thrombosis, and red blood cell dysfunction, which contribute to COVID-19 disease severity [11]. Also, Ntyonga-Pono confirmed that oxidative stress is a strong contributor to COVID-19 infections [43]. Cecchini and Cecchini suggested that COVID-19 infection pathogenesis is a consequence of oxidative stress and after that, cytokine storm and coagulopathy [44]. Also, they suggested that virus prompts oxidative stress but at the same time facilitates the nuclear translocation of Nrf2 with subsequent expression of HO-1, a protective enzyme against oxidative injury in the human alveolar epithelial cells. Our results, as well as results of previous studies, confirmed the connection between the severity of the disease; present comorbidities, such as elderly with diabetes, hypertension, and cardiovascular diseases; and elevated oxidative stress. In these patients, the viral infection will increase this stress, giving us one possible explanation of the severity of COVID-19 in these categories of patients [45]. With regard to the abovementioned, the oxidative stress is an important factor influencing the success or failure of the response to virus infection. The latest data about the role of oxidative stress in COVID-19 infection support the recommendation of antioxidant supplementation as a useful strategy against COVID-19. Several nutraceuticals have a proven ability of immune-boosting, antiviral, antioxidant, and anti-inflammatory effects. These include Zn, vitamin D, vitamin C, curcumin, cinnamaldehyde, probiotics, selenium, lactoferrin, quercetin, and other polyphenols. Grouping some of these phytonutrients in the right combination in the form of a food supplement may help to boost the immune system, prevent virus spread, preclude the disease progression to the severe stage, and further suppress the hyper inflammation providing both prophylactic support and therapeutic support against COVID-19 [46]. The literature clearly highlights the pharmacological activities of polyphenols from plants, that is, antioxidant, anti-inflammatory, anticancer, antibacterial, antifungal, and antiviral. Luteolin, daidzein, apigenin, amentoflavone, quercetin, epigallocatechin, epigallocatechin gallate, and gallocatechin gallate show antiviral activity through inhibition of the proteolytic activity of SARS-CoV 3C-like protease, which plays a key role for the viral replication [21].

The main limitations of our study are the presence of confounding factors (such as hypertension and obesity) that could influence the levels of oxidative stress parameters other than SARS-CoV2 infection. But given the fact that exclusion of all these factors could lead to bias regarding the predictive potential of each following parameter, we overcame this by using linear regression analysis. Also, the influence of different treatment and oxygenation types was not investigated and taken into consideration; also as one of confounding factors, however, most of the study population in our study was treated with the same type of treatment, differing just in oxygenation management.

We are sure that SARS-CoV2, probably like other RNA viruses, can trigger oxidative stress [44, 45] by disturbing the main reactive oxygen and nitrogen species such as superoxide anion radical and nitric oxide. Based on these results, we propose a reduction of patient's level of oxidative stress by supplying them with substances that improve their antioxidant system.

## 5. Conclusion

This is the first study to investigate the circulating oxidative stress parameters in COVID-19 patients, as well as their predictive role in the disease severity and mortality. Definitely, oxidative stress could be an important factor in this viral pneumonia and the crucial pathogenic mechanism underlying the development of this respiratory disease. We confirmed that in the different severity of SARS-CoA-2 infection are differently changed levels of superoxide anion radical, nitric oxide, and catalase activity.

Furthermore, hypertension, anosmia, and ageusia, as well as the duration of ICU stay, were identified as predictors of COVID-19 severity, while the presence of dyspnea and increased heart rate on admission were predictors of COVID-19 mortality.

## Figures and Tables

**Table 1:** Basic demographic characteristics and presence of comorbidity in the study population (*n* = 127). Data are presented as frequency (%) from the total number of participants.

Variables	Degree of COVID19 infection (*n* = 127)			
	Mild	Moderate	Severe	*p* values
Gender (M/F)	11/6 (12.6%/15%)	27/13 (31%/32.5%)	49/21 (56.3%/52.5%)	*p* = 0.903
Smoking (no/yes)	13/4 (11.8%/23.5%)	34/6 (30.9%/35.3%)	63/7 (57.3%/41.2%)	*p* = 0.318
Comorbidity (no/yes)	7/10 (14.3%/12.8%)	24/16 (49%/20.5%)	18/52 (36.7%/66.7%)	*p* = 0.002^a,b,c^
Hypertension (no/yes)	8/9 (13.3%/13.4%)	29/11 (48.3%/16.4%)	23/47 (38.3%/70.1%)	*p* ≤ 0.001^a,b,c^
Diabetes mellitus (no/yes)	11/6 (11.5%/19.4%)	34/6 (35.4%/19.4%)	51/19 (53.1%/61.3%)	*p* = 0.193
Obesity (no/yes)	12/5 (11.3%/23.8%)	36/4 (34%/19%)	58/12 (54.7%/57.1%)	*p* = 0.192
COPD (no/yes)	16/1 (13.2%/16.7%)	38/2 (31.4%/33.3%)	67/3 (55.4%/50%)	*p* = 0.957
Malignant disease (no/yes)	16/1 (13%/25%)	39/1 (37.1%/25%)	68/2 (55.3%/50%)	*p* = 0.782

Statistical significance was established by chi square test (*X*^2^ test) as follows: ^a^mild vs. moderate; ^b^mild vs. severe; ^c^moderate vs. severe.

**Table 2:** Characteristic symptoms of SARS-CoV19 infection in the study population (*n* = 127). Data are presented as frequency (%) from the total number of participants.

Variables	Degree of COVID19 infection (*n* = 127)			
	Mild	Moderate	Severe	*p* values
Elevated body temperature (no/yes)	3/14 (18.8%/12.6%)	8/32 (50%/28.8%)	5/65 (31.3%/58.6%)	*p* = 0.008^a,b,c^
Cough (no/yes)	3/14 (8.3%/15.4%)	19/21 (52.8%/23.1%)	14/56 (38.9%/61.5%)	*p* = 0.005^a,b,c^
Anosmia (no/yes)	13/4 (12%/21.1%)	35/5 (32.4%/26.3%)	60/10 (55.6%/52.6%)	*p* = 0.550
Ageusia (no/yes)	14/3 (13%/16.7%)	35/5 (32.4%/27.8%)	59/10 (54.6%/55.6%)	*p* = 0.887
Dyspnea (no/yes)	10/7 (13.9%/12.7%)	26/14 (36.1%/25.5%)	36/34 (50%/61.8%)	*p* = 0.378
Diarrhea (no/yes)	11/6 (9.7%/42.9%)	36/4 (31.9%/28.6%)	66/4 (58.4%/28.6%)	*p* = 0.002^a,b,c^

Statistical significance was established by chi square test (*X*^2^ test) as follows: ^a^mild vs. moderate; ^b^mild vs. severe; ^c^moderate vs. severe.

**Table 3:** Respiratory and cardiovascular symptoms of SARS-CoV19 infection in study population (*n* = 127). Data are presented as mean and standard deviation in all study groups.

Severity of COVID-19 infection	Respiratory symptoms									
		Age (years)	Resp rate (beats/min)	Heart rate (beats/min)	SBP (mmHg)	DBP (mmHg)	SatO_2_ (%)	PaO_2_ (mmHg)	PaCO_2_ (mmHg)	pH
Mild	Mean	59.24	24.00	88.47	139.41	81.76	92.47	72.35	44.59	549.71
	Std. deviation	14.76	4.62	11.68	15.90	9.67	5.99	25.87	12.64	316.01
	*n*	17	17	17	17	17	11	17	17	17

Moderate	Mean	49.60	19.62	82.58	127.88	77.50	93.57	77.79	44.26	694.54
	Std. deviation	16.12	1.58	7.12	13.58	8.03	6.55	38.49	9.47	182.55
	*n*	40	26	26	26	26	7	28	27	26

Severe	Mean	61.39	24.02	90.67	142.81	83.76	92.33	74.31	41.07	683.98
	Std. deviation	12.87	3.83	8.30	12.62	7.24	4.19	32.78	15.18	197.59
	*n*	70	42	42	42	42	15	51	46	43

		*p* ≤ 0.001^a,b^	*p* ≤ 0.001^a,c^	*p* ≤ 0.001^a,c^	*p* ≤ 0.001^a,b,c^	*p* = 0.010^a,b,c^	*p* = 0.596	*p* = 0.852	*p* = 0.494	*p* = 0.075

Statistical significance was established by ANOVA analysis with Tukey-B post hoc test as follows: ^a^mild vs. moderate; ^b^mild vs. severe; ^c^moderate vs. severe.

**Table 4:** Hematological markers, cardiac enzymes, and inflammatory markers in all study groups. Data are presented as mean and standard deviation in all study groups.

		Parameters											
		WBCs	Lym	Lym%	HGB	PLT	Fe	Trop	CRP	FIB	CK	CK-MB	LDH
Mild	Mean	7.47	1.36	17.50	127.43	274.45	12.29	0.00	58.65	6.12	192.00	14.04	513.35
	Std. deviation	3.47	0.69	12.57	14.56	128.62	24.53	0.00	97.44	6.21	485.70	7.74	218.50
Moderate	Mean	8.14	1.39	19.26	131.51	259.92	5.38	0.00	51.10	4.79	114.57	12.48	501.80
	Std. deviation	4.43	0.84	10.62	18.91	103.79	4.50	0.00	61.76	1.78	102.47	5.72	314.78
Severe	Mean	9.43	1.65	17.38	130.54	281.94	5.92	0.86	76.19	5.26	128.19	12.62	643.13
	Std. deviation	4.62	1.81	12.79	17.09	125.19	5.62	9.06	87.28	1.96	148.96	6.76	313.30
		*p* = 0.011^b,c^	*p* = 0.306	*p* = 0.517	*p* = 0.453	*p* = 0.405	*p* = 0.063	*p* = 0.603	*p* = 0.076	*p* = 0.112	*p* = 0.252	*p* = 0.594	*p* = 0.003^a,b,c^

Statistical significance was established by ANOVA analysis with Tukey-B post hoc test as follows: ^a^mild vs. moderate; ^b^mild vs. severe; ^c^moderate vs. severe. WBC: white blood cells (10^3^/*μ*l); Lym: lymphocytes (10^3^/*μ*l); HGB: hemoglobin (g/l); PLT: platelets (10^3^/*μ*l); Fe: iron levels (g/dl); D: dimer (mcg/ml); TropP: troponin T (ng/ml); CRP: C-reactive protein (mg/l); Fib: fibrinogen (g/l); CK and CK-MB: creatin kinase (U/l).

**Table 5:** Prooxidative and antioxidative parameters in the blood in all study groups. Data are presented as mean and standard deviation in all study groups.

Severity of COVID 19 infection		Prooxidants				Antioxidant enzymes		
		O_2_^−^ (nmol/ml)	TBARS (*μ*mol/ml)	H_2_O_2_ (nmol/ml)	NO^−^ (nmol/ml)	CAT (U/Hb × 10^3^)	SOD (U/Hb × 10^3^)	GSH (U/Hb × 10^3^)
Mild	Mean	3.50	1.34	2.30	3.03	0.79	22.49	103394.79
	Std. deviation	1.83	0.48	0.68	0.62	0.38	9.27	17473.95
	*n*	33	35	35	35	35	35	35

Moderate	Mean	4.84	1.28	2.18	3.20	0.68	19.76	96202.84
	Std. deviation	3.29	0.64	0.65	0.68	0.36	9.53	21664.02
	*n*	48	50	50	50	50	48	50

Severe	Mean	11.30	1.20	2.07	2.66	1.15	22.39	95272.71
	Std. deviation	5.66	0.71	0.44	0.45	0.90	11.19	21315.30
	*n*	31	34	34	34	34	33	34

		*p* ≤ 0.001^a,b,c^	*p* = 0.603	*p* = 0.287	*p* ≤ 0.001^b,c^	*p* = 0.001^a,b,c^	*p* = 0.363	*p* = 0.184

Statistical significance was established by ANOVA analysis with Tukey-B post hoc test as follows: ^a^mild vs. moderate; ^b^mild vs. severe; ^c^moderate vs. severe.

**Table 6:** Correlation matrix of significantly different parameters between groups of patients. Results of Pearson's correlation analysis are presented as statistical significance (*p*) and with Pearson correlation coefficient (*R*).

		Age	HTA	Anosmia	Ageusia	Dyspnea	RR	HR	SBP	DBP	PaO_2_	PaCO_2_	WBC	O_2_	NO	CAT	RTG outcome	Outcome	ICU (days)	Hospital (days)
Age	*R*																			
	*p*																			
HTA	*R*	0.569^∗∗^																		
	*p*	0.00																		
Anosmia	*R*	0.06	0.04																	
	*p*	0.49	0.63																	
Ageusia	*R*	0.05	0.03	0.969^∗∗^																
	*p*	0.61	0.77	0.00																
Dyspnea	*R*	0.180^∗^	0.13	0.12	0.10															
	*p*	0.04	0.16	0.17	0.24															
RR	*R*	0.402^∗∗^	0.367^∗∗^	0.02	0.02	0.434^∗∗^														
	*p*	0.00	0.00	0.82	0.84	0.00														
HR	*R*	0.20	0.20	−0.04	0.07	0.18	0.452^∗∗^													
	*p*	0.06	0.06	0.75	0.53	0.10	0.00													
SBP	*R*	0.646^∗∗^	0.598^∗∗^	0.21	0.235^∗^	0.257^∗^	0.493^∗∗^	0.340^∗∗^												
	*p*	0.00	0.00	0.05	0.03	0.02	0.00	0.00												
DBP	*R*	0.474^∗∗^	0.480^∗∗^	0.17	0.19	0.13	0.434^∗∗^	0.261^∗^	0.769^∗∗^											
	*p*	0.00	0.00	0.12	0.09	0.25	0.00	0.02	0.00											
PaO_2_	*R*	−0.02	−0.10	−0.11	−0.10	−0.16	−0.357^∗∗^	−0.20	−0.261^∗^	−0.257^∗^										
	*p*	0.81	0.32	0.28	0.31	0.11	0.00	0.07	0.02	0.02										
PaCO_2_	*R*	0.02	−0.09	−0.01	−0.03	−0.04	0.01	−0.15	0.06	0.00	0.08									
	*p*	0.87	0.41	0.93	0.80	0.74	0.91	0.17	0.62	0.98	0.48									
WBC	*R*	0.743^∗∗^	0.616^∗^	0.45	0.39	0.21	0.828^∗^	0.04	0.41	0.17	−0.41	−0.34								
	*p*	0.01	0.04	0.00	0.00	0.54	0.04	0.94	0.41	0.74	0.37	0.51								
O_2_	*R*	0.04	0.11	−0.10	−0.10	−0.04	0.00	0.05	0.06	−0.03	0.12	0.16	0.52							
	*p*	0.67	0.24	0.31	0.32	0.65	0.98	0.65	0.58	0.76	0.28	0.16	0.13							
NO	*R*	−0.226^∗^	−0.06	0.05	0.07	−0.15	−0.278^∗^	−0.10	−0.14	−0.02	0.229^∗^	−0.08	−0.27	−0.17						
	*p*	0.02	0.50	0.62	0.45	0.10	0.01	0.37	0.21	0.82	0.03	0.48	0.43	0.07						
CAT	*R*	0.08	−0.05	−0.03	−0.04	−0.07	0.13	0.18	0.03	0.08	−0.234^∗^	0.11	−0.30	0.15	−0.249^∗∗^					
	*p*	0.40	0.60	0.71	0.71	0.47	0.25	0.11	0.77	0.47	0.02	0.30	0.37	0.12	0.01					
RTG_outcome	*R*	0.349^∗∗^	0.18	−0.02	−0.03	0.341^∗∗^	0.354^∗∗^	0.15	0.406^∗∗^	0.267^∗^	−.314^∗∗^	−0.14	−0.39	−0.10	−0.265^∗∗^	0.18				
	*p*	0.00	0.07	0.87	0.75	0.00	0.00	0.23	0.00	0.03	0.01	0.26	0.31	0.38	0.01	0.08				
Outcome	*R*	0.341^∗∗^	0.255^∗∗^	0.08	0.09	0.246^∗∗^	0.432^∗∗^	0.418^∗∗^	0.415^∗∗^	0.365^∗∗^	−0.08	−0.06	0.65	−0.07	−0.09	0.05	0.32			
	*p*	0.00	0.00	0.38	0.33	0.01	0.00	0.00	0.00	0.00	0.44	0.60	0.00	0.47	0.37	0.62	0.00			
ICU (days)	*R*	0.231^∗∗^	0.09	−0.10	−0.11	0.08	0.367^∗∗^	0.274^∗^	0.409^∗∗^	0.21	−0.16	−0.04	−0.02	0.02	−.243^∗∗^	0.13	0.17	0.439^∗∗^		
	*p*	0.01	0.30	0.25	0.23	0.38	0.00	0.01	0.00	0.06	0.13	0.73	0.96	0.83	0.01	0.15	0.08	0.00		
Hospital (days)	*R*	0.287^∗∗^	0.13	−0.14	−0.16	0.10	0.19	0.06	0.248^∗^	0.06	0.06	−0.05	0.21	−0.08	−.185^∗^	−0.02	0.336^∗∗^	0.11	0.743^∗∗^	
	*p*	0.00	0.17	0.12	0.07	0.28	0.08	0.61	0.02	0.58	0.59	0.64	0.53	0.42	0.05	0.82	0.00	0.22	0.00	

Pearson's correlation. RR: respiratory rate; HR: heart rate; SBP: systolic blood pressure; DBP: diastolic blood pressure; WBC: white blood cells; O2: superoxide anion radical; NO: nitric oxide; CAT: catalase; ICU: intensive care unit.

**Table 7:** Linear regression analysis of candidate predictors for dependent variable—severity of COVID-19 infection (mild, moderate, or severe form of SARS-CoV-2 infection).

	Dependent variable: severity of COVID-19 infection				
	Unstandardized coefficients (*B*)	Std. error	Standardized coefficients (beta)	*t*	*p*
Age	0.007	0.006	0.133	1.111	0.270
Male gender	−0.022	0.146	−0.014	−0.152	0.879
Present HTA	0.322	0.124	0.225	2.598	0.011
Elevated body temperature	0.310	0.186	0.144	1.662	0.099
Anosmia	−1.621	0.696	−0.811	−2.328	0.022
Ageusia	1.601	0.712	0.783	2.247	0.026
Cough	−0.015	0.160	−0.010	−0.096	0.924
Dyspnea	0.101	0.141	0.070	0.714	0.477
RR	0.005	0.026	0.023	0.178	0.859
HR	0.012	0.010	0.143	1.172	0.245
SBP	0.008	0.009	0.148	0.845	0.401
DBP	0.006	0.016	0.065	0.370	0.712
PaO_2_	0.001	0.003	0.042	0.365	0.716
WBC	0.447	0.251	1.040	1.781	0.217
O_2_-	0.087	0.012	0.565	7.330	≤0.001
H_2_O_2_	−0.069	0.098	−0.056	−0.700	0.485
TBARS	−0.055	0.118	−0.045	−0.464	0.644
NO-	−0.087	0.093	−0.074	−0.932	0.353
CAT	0.175	0.098	0.141	1.780	0.078
Hospital (days)	−0.098	0.084	−0.641	−1.163	0.365
ICU (days)	0.025	0.013	0.191	1.989	0.049

RR: respiratory rate; HR: heart rate; SBP: systolic blood pressure; DBP: diastolic blood pressure; WBC: white blood cells; O_2_^−^: superoxide anion radical; H_2_O_2_: hydrogen peroxide; CAT: catalase; ICU: intensive care unit.

**Table 8:** Linear regression analysis of candidate predictors for dependent variable—outcome of COVID-19 infection (positive or negative outcome).

Potential predictors	Dependent variable: outcome of COVID-19 infection				
	Unstandardized coefficients (*B*)	Std. error	Standardized coefficients (beta)	*t*	*p*
HTA	0.049	0.113	0.070	0.436	0.664
DM	0.007	0.085	0.008	0.078	0.938
Obesity	−0.006	0.092	−0.006	−0.060	0.953
Smoking	−0.052	0.091	−0.050	−0.568	0.571
Temp	0.062	0.100	0.059	0.621	0.536
Anosmia	0.074	0.089	0.076	0.836	0.405
Cough	0.105	0.074	0.134	1.422	0.158
Dyspnea	0.179	0.063	0.252	2.826	0.006
Diarrhea	0.022	0.099	0.020	0.224	0.823
RR	0.022	0.012	0.224	1.872	0.065
HR	0.011	0.005	0.262	2.432	0.017
SBP	0.005	0.004	0.190	1.207	0.231
DBP	0.004	0.007	0.079	0.518	0.606
PaO_2_	0.001	0.001	0.115	1.115	0.268
O_2_^−^	−0.007	0.007	−0.096	−0.975	0.332
H_2_O_2_	−0.081	0.059	−0.139	−1.376	0.172
CAT	0.018	0.059	0.032	0.312	0.756

## Data Availability

All data are available upon request.
